# An extended cluster expansion for ground states of heterofullerenes

**DOI:** 10.1038/s41598-017-16469-0

**Published:** 2017-11-24

**Authors:** Yun-Hua Cheng, Ji-Hai Liao, Yu-Jun Zhao, Xiao-Bao Yang

**Affiliations:** 1 Department of Physics, South China University of Technology, Guangzhou, 510640 People’s Republic of China; 2 Key Laboratory of Advanced Energy Storage Materials of Guangdong Province, South China University of Technology, Guangzhou, 510640 P. R. China

**Keywords:** Density functional theory, Density functional theory, Method development, Method development, Structure prediction

## Abstract

It is challenging to determine the ground states of heterofullerenes due to the numerous isomers. Taking the C_60-*n*_B_*n*_ heterofullerenes (1 ≤ *n* ≤ 4) as an example, our first-principles calculations with the isomer enumeration present the most stable structure of C_57_B_3_, which is energetically favored by 0.73 eV than the reported counterpart. It was difficult to conduct the enumeration for the isomers with *n* beyond 4 because of the expensive first-principle calculations. Here, we propose a nomenclature to enhance structural recognition and adopt an extended cluster expansion to describe the structural stabilities, in which the energies of the heterofullerenes with various concentrations are predicted by linear combination of the multi-body interactions. Unlike the conventional cluster expansion, the interaction parameters are derived from the enumeration of C_60-*n*_B_*n*_ (*n* = 1~4), where there are only 4 coefficients to be fitted as a function of composition for the consideration of local bonding. The cross-validation scores are 1~2 meV per atom for both C_55_B_5_ and C_54_B_6_, ensuring the ground states obtained from our model are in line with the first-principles results. With the help of the structural recognition, the extended cluster expansion could be further applied to other binary systems as an effective complement to the first-principle calculations.

## Introduction

Since the discovery in 1985^1^, the C_60_ fullerene has attracted great attentions due to various potential applications with its unique structure-dependent properties. The cage of C_60_ fullerene, with the size which is large enough to be observed by transmission electron microscopy or scanning probe methods^2,3^, is likely to keep stable when built into molecular circuits as semiconductor materials^4–7^. Doping has been adopted as the conceivable way to alter its charge distribution and then tune the optical, electronic and magnetic properties in the solid state^8–10^, including the way of adding exohedral, endohedral and substitutional atoms^11^. As neighbors to carbon in the Periodic Table, boron and nitrogen with similar atomic radius are the popular choices as the heteroatoms for the substitution of one or more of the carbon atoms^12–14^. The C_60-*n*_B_*n*_ heterofullerenes with 1 ≤ n ≤ 6 were produced by Laser vaporization of a graphite pellet containing boron nitride powder^15^, which indicated that boron doped C_60_ cage still appeared to be particularly stable. During the synthesis and characterization of C_59_N^16^, C_59_N in the vapor phase was found existing in monomer form as a molecular free radical^17^, where the single C_59_N heterofullerene molecule could be used as a new molecular rectifier in a double-barrier tunnel junction via the single electron tunneling effect^18^.

Theoretically, Kurita *et al*. found that the molecular structures of C_59_B and C_59_N maintained the cage of C_60_, which was distorted due to a large-size dopant such as Sulfur^19^. In addition to the calculation of C_59_B by the first-principles method^20^, the ground state geometries of C_60-*n*_N_*n*_ and C_60-*n*_B_*n*_ for 2 ≤ *n* ≤ 8 were screened using semiempirical MNDO, AM1, PM3, and *ab initio* methods^21^. As C_48_B_12_ and C_48_N_12_ are promising components for molecular rectifiers^22^, Garg *et al*.^23^ reported a detailed study of structural, electronic and vibrational properties of B-doped heterofullerenes (C_60-*n*_B_*n*_, for *n* = 1~12) based on *ab initio* calculations, concluding that the maximum number of boron atoms in a pentagon/hexagon ring was one/two. In general, it is difficult to determine the ground state of heterofullerenes due to two main obstacles: (i) only small amount of isomers for given compositions were considered; (ii) the optimized heterofullerenes largely depend on the initial geometry of numerous possible isomers^24^. Hence, there is still a lack of theoretical studies to search the energetically-preferred structures of heterofullerenes.

In this paper, we perform a systematic investigation of C_60-*n*_B_*n*_ (*n* = 1~6) based on the first-principles calculations with the congruence check, in which the structure recognition is achieved by a uniform numbering scheme for C_60_. Furthermore, an extended cluster expansion is proposed to estimate the total energies with all the possible pair, three-body, and four-body interactions derived from the enumeration of C_60-*n*_B_*n*_ (*n* = 1~4), however, there are only four coefficients to be fitted for the consideration of composition. We determine the ground state structures of C_55_B_5_ and C_54_B_6_ as confirmed by the first-principles calculations, indicating the possible application to other alloy systems.

## Structure recognition

**Figure 1:**
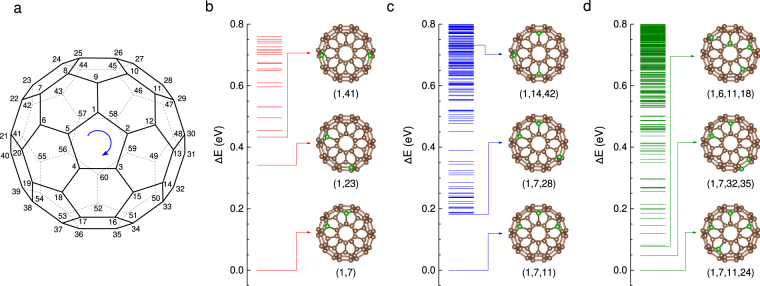
C_60_ fullerene cage with the systematic numbering scheme^25^ and the isomers energy relative to the lowest energy for C_60-*n*_B_*n*_ (2 ≤ n ≤ 4). (**a**) Systematic numbering scheme. Any atom in the C_60_ fullerene (the coordinates are listed in Supplementary Table S1) has its unique sequence number (SN). The arrow indicates the direction of the numbering commencement. (**b**) The energies of C_58_B_2_ isomers. The lowest, the 2^nd^ and 3^rd^ lowest energetic structure of C_58_B_2_, denoted by (1, 7), (1, 23) and (1, 41), respectively are shown. (**c**) The energies of C_57_B_3_ isomers. The cage with the lowest and the 2^nd^ lowest energy denoted by (1, 7, 11) and (1, 7, 28), respectively are shown. The cage denoted by (1, 14, 42) is the previously reported^23^ ground state of C_57_B_3_ which has a higher energy of 0.73 eV than the global minimal state (1, 7, 11). (**d**) The energies of C_56_B_4_ isomers. The global minimal energetic structure, the 2^nd^ and 3^rd^ lowest energetic cage, denoted by (1, 7, 11, 24), (1, 7, 32, 35) and (1, 6, 11, 18), respectively, are shown. In (**b**), (**c**) and (**d**), we only show the relative energies lower than 0.8 eV.

We adopt the systematic numbering scheme recommended by the IUPAC^25^ to identify the vertices of the C_60_ cage, as shown in Fig. 1a. Any atom in the C_60_ fullerene cage (the coordinates are listed in Supplementary Table S1) has its unique sequence number (SN). An isomer of C_60-*n*_B_*n*_ heterofullerenes is denoted by an index consisting of the ascending ordered SNs of the substituted vertices, *i.e*.$\,({\sigma }_{1},{\sigma }_{2},\ldots ,{\sigma }_{n})$ which is called structural index (SI). According to the congruence check, the total number of the C_60-*n*_B_*n*_ (*n* ≤ 4) heterofullerenes isomers is 4,517, which is only about 1% of the corresponding combination number (see Supplementary Table S2). All the total energies of these candidates are obtained by the first-principles calculations (details in Supplementary Information), where all the calculated structures are fully relaxed without any symmetry constraint. The ground state structures with the energy profiles are shown in Fig. 1. There are 23 different isomers (shown in Table 1) for C_58_B_2_, which is in agreement with earlier calculations^23,26,27^. As shown in Fig. 1b, the global minimum structure of C_58_B_2_ is the cage with two boron atoms at the opposite sites of a hexagon ring. The ground states of C_57_B_3_ and C_56_B_4_, shown in Fig. 1c and d, respectively, have the similar pattern that all boron atoms are at the opposite vertices of the hexagon rings adjacent to each other, where there are no more than two boron atoms on a hexagon ring. The ground state structures of C_58_B_2_ and C_56_B_4_ are in agreement with those previous calculations^21,23^. However, the total energy of the most stable C_57_B_3_ is 0.73 eV lower than that of the one proposed by Garg *et al*.^23^, which ranks 126^th^ according to our enumeration of the 303 isomers as shown in Fig. 1c.

**Table 1:** The 23 isomers of C_58_B_2_.

SI	*E* (eV)	SI	*E* (eV)	SI	*E* (eV)	SI	*E* (eV)
(1, 7)	0.00	(1, 3)	0.53	(1, 32)	0.67	(1, 24)	0.74
(1, 23)	0.34	(1, 16)	0.59	(1, 57)	0.70	(1, 15)	0.74
(1, 41)	0.43	(1, 33)	0.61	(1, 9)	0.71	(1, 14)	0.75
(1, 50)	0.45	(1, 56)	0.65	(1, 35)	0.71	(1, 34)	0.76
(1, 52)	0.49	(1, 49)	0.65	(1, 31)	0.71	(1, 2)	1.19
(1, 60)	0.53	(1, 13)	0.67	(1, 6)	0.72		

The structural indexes (SIs) of C_58_B_2_ isomers are sorted in the order of ascending energy (columns titled by *E*) relative to that of the isomer denoted by (1, 7), which is the minimal energetic structure of the C_58_B_2_ heterofullerenes.

Buckminsterfullerene, *i.e*., the only isomer fulfilling the isolated pentagon rule (IPR)^28^, is a spherical molecule with 60 carbon atoms at vertices, containing 32 faces including 20 hexagons and 12 pentagons where no pentagon shares a vertex^29^. Treating C_60_ as a semiregular polyhedron and considering all the symmetry operations represented by the 120 symmetry matrices (SMs), we obtain a 60 × 120 matrix called numbering matrix (NM) (available in the Supplementary Dataset file), in which the *n* ^th^ row lists the coincident atoms for the *n* ^th^ atom under all the symmetry operations and the *n* ^th^ column contains the corresponding coincident atoms for all the 60 atoms under the operation of the *n* ^th^ SM.

Herein, based on the NM of C_60_, we propose a nomenclature for the C_60-*n*_B_*n*_ heterofullerenes. The flow chart of our structure recognition method is shown in Supplementary Fig. S1. The detailed information of the structure recognition method is available in the Supplementary Information. We have deduced all the SIs of the inequivalent structures of C_60-*n*_B_*n*_ heterofullerenes for 2 ≤ *n* ≤ 10, the numbers of the SIs are listed in Supplementary Table S2, which is in good agreement with the previous results^24,30,31^. The inequivalent structures can also be singled out by our recently developed structure recognition method^32^. Note that our nomenclature is derived from the symmetry operation matrices, which can be obtained according to the coordinates of the system with the corresponding symmetry operations. Thus, this nomenclature can be extended for the C_60_ with non-IPR isomers, as well as the larger fullerenes e.g. C_70_ and C_82_.

## Extended cluster expansion method

Combined with the isomer enumeration and the first-principles calculations, we have determined the ground state structures of C_60-*n*_B_*n*_ heterofullerenes with 2 ≤ n ≤ 4. For those heterofullerenes with higher boron concentration, we can enumerate all the isomers by the recognition method discussed above. However, it will be over expensive to search the ground state structures with the first-principles calculations, because there are 45,718, 418,470, 3,220,218, 21,330,558, 123,204,921 and 628,330,629 isomers for C_55_B_5_, C_54_B_6_, C_53_B_7_, C_52_B_8_, C_51_B_9_ and C_50_B_10_, respectively. Analogue to the conventional cluster expansion(CE)^33^, an extended cluster expansion (ExCE) method is discussed aiming at this problem in the following.

As is known, the CE method is an efficient tool for studying structural properties of any binary structures over a wide range of concentrations^34–38^, parameterizing the total energy for any given configuration of A_x_B_1−x_ (0 ≤ *x* ≤ 1) to avoid the expensive cost of the first-principles calculations. The enthalpy of formation for a certain configuration $\mathop{s}\limits^{\rightharpoonup }$ is described exactly by a set of multi-body interaction parameters ***J***_***i***_ combined as the form of an Ising-like Hamiltonian^37^, which is often approximated as a polynomial function of occupation variables,

1 $${\rm{\Delta }}{H}_{CE}(\mathop{s}\limits^{\rightharpoonup })=\sum _{\alpha }{m}_{\alpha }{J}_{\alpha }\prod _{i\in s^{\prime} }{s}_{i}$$

where the summation is over all the non-equivalent clusters, a set of sites *i* denoted by *α*, and the average is taken over all the clusters *α* that are equivalent to *α* by symmetry. The coefficients are defined as effective cluster interaction (ECI) parameters, and *m*_*α*_ is the number of the clusters equivalent to *α*.

In general, the total energies of given configurations are described by the combine of single-atom contributions, pair interactions and multi-body interactions, which are expected to gradually converge as more interactions are considered. However, fitting with larger number of parameters will be also time expensive. To balance the accuracy and efficiency of CE method, the number of effective multisite interactions can be greatly reduced^39^, while the combinations of possible effective interactions result in another global optimization. Herein, we attempt to derive the multi-body interactions taking account of the total impurity concentration and fit the total energy with fewer parameters for higher accuracy.

An isomer of C_60-*n*_B_*n*_ heterofullerenes, taking the carbon atoms as the background, can be viewed as a cluster of boron atoms denoted by $\,({\sigma }_{1},{\sigma }_{2},\cdots ,{\sigma }_{n})$. We suppose the total energy be attributed to all the subclusters, which are enumerated for fitting the total energy. Firstly, there is only one isomer for C_59_B, therefore the energy difference of C_59_B relative to C_60_ is *E*_1_ = *E*_*tot*_ − *E*_0_ = 3.39 eV, where *E*_*tot*_ and *E*_0_ are the total energy of C_59_B and C_60_, respectively. The energy difference *E*_1_ is responsible for the reaction heat when one C atom is substituted by one boron atom, which can be considered as the single-atom contribution in the expansion. For a C_58_B_2_ isomer whose subclusters are two equivalent boron single dopants, the environment of each boron atom is different from that of C_59_B, thus the energies can be expressed as *E*_0_ + 2*c*_1_ *E*_1_ with the coefficient *c*_1_ as a function of boron concentration. Based on all the energies of C_58_B_2_ isomers, we fit this coefficient *c*_1_ to be 0.980 with the average deviation of 0.208 eV. Similar to the CE method, the fitting quality is determined by the cross-validation (CV) score^39^,

2 $$CV=\sqrt{\frac{1}{n}\sum _{i=1}^{n}{({E}_{i}^{DFT}-{\hat{E}}_{i})}^{2}}$$

where ${E}_{i}^{DFT}$ and ${\hat{E}}_{i}$ denote the DFT calculated and predicted energy of a particular structure *i*. The deviation of ${E}_{i}^{DFT}$ from ${\hat{E}}_{i}$ is taken as the interactive energy of the corresponding boron cluster. For C_58_B_2_, The 23 fitting deviations are the B-B interactions in the 23 isomers, respectively.

**Figure 2:**
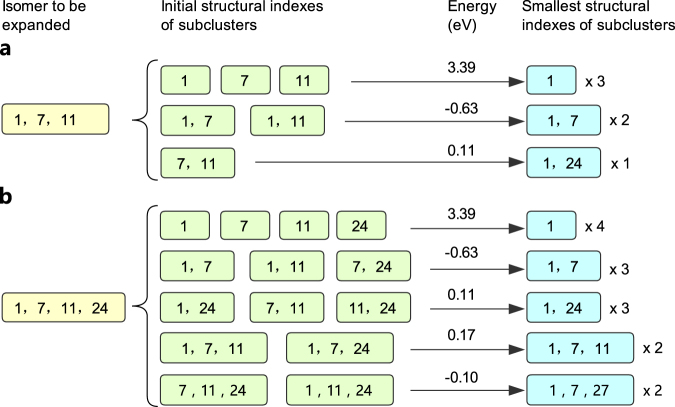
Examples for the cluster expansion. (**a**) Expansion of (1, 7, 11). (**b**) Expansion of (1, 7, 11, 24). The initial structural indexes (SIs) are obtained simply from all possible combinations for the numbers from the SI of the isomer to be expanded. The smallest SIs are obtained by our structure recognition method. The energy values shown are the fitting errors which are supposed to be the energetic contributions of the corresponding subclusters. The numbers after multiple signs indicate the numbers of equivalent subclusters in cluster expansion.

Similarly, the total energy of a C_57_B_3_ isomer denoted by $\,\mathop{\sigma }\limits^{\rightharpoonup }$, is contributed by three single dopants and three pair interactions. For example, the isomer of C_57_B_3_ denoted by (1, 7, 11) can be expanded as 6 clusters including 3 singles and 3 pairs. The initial SIs of the subclusters are listed in Fig. 2a along with their smallest SIs by our recognition method. Apart from the singles, the isomer denoted by (1, 7, 11) has the 3 subclusters denoted by (1, 7), (1, 7) and (1, 24). Hence we express the total energy as $\,{E}_{0}+3{c}_{1}{E}_{1}+{c}_{2}\sum _{\alpha }{E}_{2}^{\alpha }$, where ${E}_{2}^{\alpha }$ denotes the B-B interaction for any boron pair as a subcluster of $\,\mathop{\sigma }\limits^{\rightharpoonup }$. The coefficients *c*_1_ and *c*_2_ are fitted to be 0.972 and 0.782 respectively, and the average deviation is 0.097 eV. The 303 fitting deviations are further taken as the 303 3-body interactive energies, respectively. For a C_56_B_4_ heterofullerene, the total energy is contributed by 4 single dopants, 6 pair interactions, and 4 triplet interactions. For example, the subclusters from the expansion of the isomer of C_56_B_4_ denoted by (1, 7, 11, 24) are listed in Fig. 2b. Analogously, we obtain the 4,190 4-body interactions when the energies of all C_56_B_4_ isomers are fitted by *E*_0_, 3*E*_1_, the 2-body and 3-body interactions, in which the average fitting deviation is 0.065 eV and the coefficients *c*_1_ *,c*_2_ and *c*_3_ are 0.966, 0.690 and 0.640, respectively.

**Figure 3:**
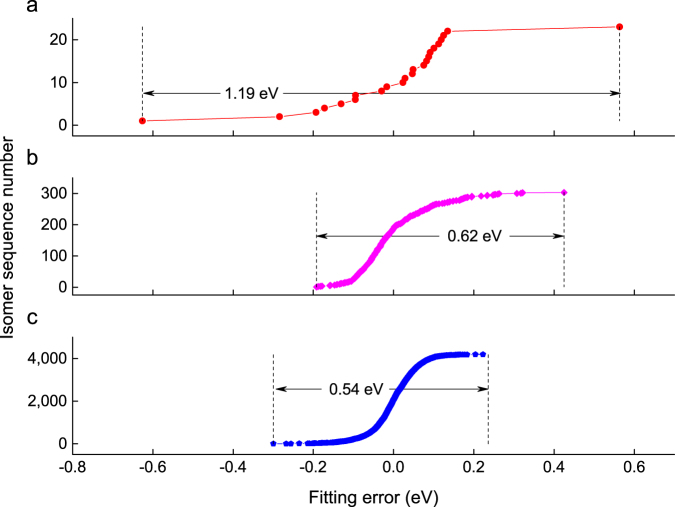
Distribution of the fitting errors of C_58_B_2_, C_57_B_3_ and C_56_B_4_. (**a**) The energetic contribution of boron pairs. (**b**) The interactive energy of boron triplets. (**c**) The interactive energy of boron quadruplets. The y-axis represents the sequence numbers for isomers when sorted by their fitting errors in ascending order. The ranges of the x-axis are the same so that we can easily compare the distribution ranges of the fitting errors of different interactions marked by figures between pairs of arrows.

As shown above, the coefficients reflect the boron atom’s concentration and the fitting deviations are attributed to the multi-body interactions. The fitting coefficients and CV scores for C_58_B_2_, C_57_B_3_ and C_56_B_4_ are listed in Table 2. It shows that the introducing of multi-body interactions will improve the accuracy of cluster expansion and the interactions will decrease as boron dopants increasing. For example, the coefficients *c*_1_ is from 0.980 to 0.966, and *c*_2_ is from 0.782 to 0.690. Fig. 3 shows the statistical distributions for the 2-body, 3-body and 4-body interactions. Compared to the 3-body and 4-body interactions, the 2-body interactions distribute in a wider range. The 4-body interactions exhibit the similar characteristics of normal distribution around zero point. It can be inferred that the interactions of 2-body and 3-body are much more important than that of 4-body or other multi-body interactions, hence the fitting will also reach a rather good convergence even if only 2-body 3-body interactions are considered.

**Table 2:** Cross-validation (CV) score versus fitting cutoff.

C_60-*n*_B_*n*_	Fitting cutoff	*c*_1_	*c*_2_	*c*_3_	*c*_4_	CV (eV)
C_58_B_2_	1	0.980				0.208
C_57_B_3_	2	0.972	0.782			0.097
C_56_B_4_	3	0.966	0.690	0.640		0.065
C_55_B_5_	2	0.928	0.200			0.106
C_55_B_5_	3	0.959	0.594	0.416		0.064
C_55_B_5_	4	0.960	0.585	0.429	0.132	0.060
C_54_B_6_	2	0.919	0.160			0.148
C_54_B_6_	3	0.963	0.592	0.397		0.125
C_54_B_6_	4	0.963	0.587	0.399	0.031	0.124

Fitting cutoff refers to the number of atoms in the largest subcluster used for the fitting. We perform three kinds of fittings for C_55_B_5_ and C_54_B_6_ in which the largest subcluster are pair, triplet and quadruplet, respectively.

Herein, we propose an extended cluster expansion for the C_60-*n*_B_*n*_ heterofullerene, where the energy of isomer denoted by $\,\mathop{\sigma }\limits^{\rightharpoonup }$ is expressed as

3 $${E}_{DFT}(\mathop{\sigma }\limits^{\rightharpoonup })=\hat{E}(\mathop{\sigma }\limits^{\rightharpoonup })+{E}_{n}$$

where $\,{E}_{DFT}(\mathop{\sigma }\limits^{\rightharpoonup })$ and $\hat{E}(\mathop{\sigma }\limits^{\rightharpoonup })$ are the DFT calculated energy and the predicted energy, respectively, and *E*_*n*_ denotes the fitting deviation which is supposed to be responsible for the n-body interaction of the boron cluster denoted by $\,\mathop{\sigma }\limits^{\rightharpoonup }$. The predicted energy is as follows,

4 $$\hat{E}(\mathop{\sigma }\limits^{\rightharpoonup })={E}_{0}+\sum _{i=1}^{n-1}{c}_{i}{E}_{i}$$

where the summation runs over all possible sizes of the subclusters of $\,\mathop{\sigma }\limits^{\rightharpoonup }$. The first term *E*_0_ represents the energy of C_60_, and *E*_*i*_ denotes the total effective energy contribution from all the clusters with *i* heteroatoms, as is expressed as below

5 $${E}_{i}=\sum _{\alpha }{E}_{i}^{\alpha }$$

where the summation is over all those subclusters consisting of *i* heteroatoms, *i.e*. $1\le \alpha \le {C}_{n}^{i}$. Different from the conventional CE, the multi-body interactive energies *E*_*i*_ apart from *E*_*n*_, should be multiplied by different combination coefficients *c*_*i*_ before they make contribution to the total energy of the C_60-*n*_B_*n*_ heterofullerene cages, where the coefficients are obtained by fitting the DFT-calculated energies of the selected C_60-*n*_B_*n*_ heterofullerenes with those *E*_*i*_ for *i* < *n*. To balance the accuracy and computation cost, we set 4 as the maximum value of the summing index in Eq. (4) for the cages of C_60-*n*_B_*n*_ where *n* ≥ 5.

**Figure 4:**
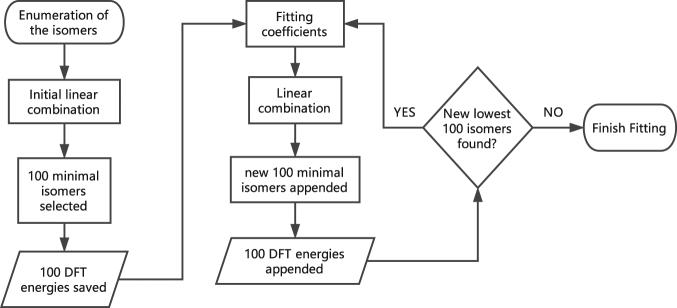
Flowchart for the extended cluster expansion (ExCE) method. The selection of the coefficients for the initial linear combination have a certain arbitrariness, which may bring about the different 100 lowest energy isomers for the first fitting iteration but not affect the final convergence and the searching for the putative ground state isomer. Here, we set 100 as the number of the new added isomers for each iteration, which may differ for different iteration and alloy systems.

We show the flow chart of our method in Fig. 4 and make a detailed description for the process in searching the ground state structures for the C_55_B_5_ cage.

1. Generate the SIs of all the C_55_B_5_ isomers (45,718 in all), list all the subclusters of these SIs and calculate the total energies of the isomers by Eq. (3), where the combination coefficients for the interaction of singles, pairs and triplets are from the fitting of the energies of C_56_B_4_, and the coefficient for quadruplet-body interaction is 1.
2. Choose the 100 minimum energetic structures and calculate their total energies (saved in ${E}_{i}^{DFT}$) using the first-principles calculations.
3. Retain the corresponding coefficients with the total energies from the first-principles calculations. Use the coefficients to calculate the total energies of all the isomers by Eq. (3).
4. Apart from those selected before, select the 100 minimum energetic structures and calculate their total energies (appended to ${E}_{i}^{DFT}$) using DFT.
5. Fit the energies of the structures, which has been selected until now, by Eq. (3), and update the corresponding coefficients.
6. Use the coefficients to calculate the total energies of all the isomers.
7. Check whether the latest DFT calculation brings about new structures whose energies are among the minimum 100 ones in ${E}_{i}^{DFT}$ If it does, repeat the steps 4 to 7, otherwise, break the process.

The fitting ultimately reaches a rather reasonable convergence after several hundreds of structures with the lowest predicted energy are calculated. Supplementary Table S3 and Table S4 shows the variations of coefficients for C_55_B_5_ and C_54_B_6_, respectively, as a function of the number of isomers calculated by the first-principles method. Note that the coefficients of *c*_2_ and *c*_3_ are around 0.6 and 0.4 respectively, while the coefficient *c*_4_ is approaching zero. Similar to the conventional CE, the energy of interatomic bonds is usually dominated by short-range interactions^39^. On the other hand, enormous interactions would be introduced when we use the C_60-*n*_B_*n*_ with higher boron concentration for cluster expansion, which will be expensive in computational cost. Note that any other binary systems can be similarly searched by the extended cluster expansion, where the appropriate cutoff of the size of the subcluster should be carefully made to balance of the accuracy and computation cost. The nomenclature and extended cluster expansion can be also applied for the ternary systems, where different atoms are distinguished from the candidates found in the binary systems.

## Application to C_55_B_5_ and C_54_B_6_

**Figure 5:**
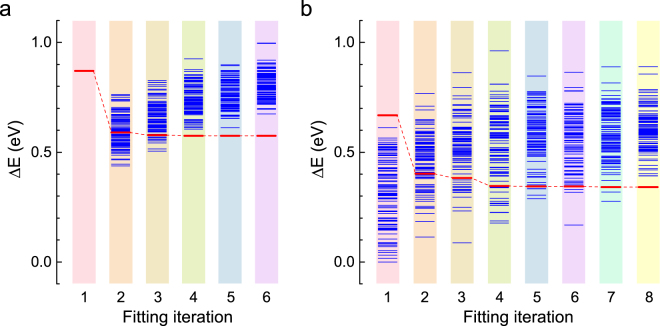
Calculated energies of the isomers in the fitting process. For each fitting iteration, the new found 100 C_55_B_5_ and C_54_B_6_ isomers with the lowest energies are shown in (**a**) and (**b**), respectively. With the fitting iteration moving forward, the numbers of the fitted isomers have an increase of 100 per iteration, reaching 600 and 800 finally in (**a**) and (**b**), respectively. In (**a**) and (**b**), all the energies are relative to the corresponding minimum ones. Only the energies of those newly added isomers for each iteration are shown. The solid line linked by the dashed lines represents the 100^th^ lowest energy of the current fitting iteration.

According to the structure recognition, there are 45,718 and 418,470 inequivalent structures for C_55_B_5_ and C_54_B_6_, respectively. Using the method discussed above and following those steps, we have made a prediction for the ground state structures of C_55_B_5_ and C_54_B_6_, where the energy profiles are shown in Fig. 5 (detailed in Supplementary Table S5). The optimized fitting coefficients of C_55_B_5_ are adopted for the initial combination coefficients for C_54_B_6_. As the fitting steps move forward, most of the energies of the newly added isomers in each fitting iteration gradually increase. However, the 100^th^ lowest energy of the fitting iteration decreases rapidly and eventually converges, after 6/8 fitting iterations for C_55_B_5_/C_54_B_6_. The minimum energetic isomers for both C_55_B_5_ and C_54_B_6_ emerge in the first iteration. The optimized fitting coefficients were obtained for C_55_B_5_/C_54_B_6_ after the energies of the selected 600/800 isomers are calculated and fitted, for which the results are listed in Table 2. The CV score of the final fitting is 0.064/0.124 eV for C_55_B_5_/C_54_B_6_, and the largest deviation of total energy between ExCE method and DFT calculations is 0.192/0.403 eV for C_55_B_5_/C_54_B_6_, indicating that the fitting energies are in good agreement with the DFT calculations. For both C_55_B_5_ and C_54_B_6_, the coefficients *c*_1_ are close to 1, implying that single boron atom does make an important contribution despite of the concentration. The coefficient *c*_4_ is much smaller and the quadruplet interactions play a trivial role in the ExCE calculations, since the fitting will be in good accuracy when the pair and triplet interactions are considered in the energy predications of C_60-*n*_B_*n*_ for n ≥ 5. This is consistent with the above assumption that the energy of interatomic bonds should be usually dominated by short-range interactions.

**Figure 6:**
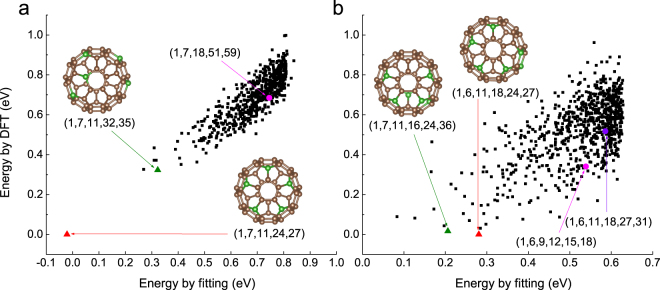
Energy predicted by ExCE versus DFT calculated energy. (**a**) The predicted and DFT calculated energies for C_55_B_5_ isomers. The putative ground state and the isomer with the 2^nd^ lowest energy for C_55_B_5_ are denoted by (1, 7, 11, 24, 27) and (1, 7, 11, 32, 35), respectively. The ground state of C_55_B_5_ reported previously^23^ is marked as (1, 7, 18, 51, 59). (**b**) The predicted and the DFT calculated energies for C_54_B_6_ isomers. The ground state and the isomer with the 2^nd^ lowest energy for C_54_B_6_ are denoted by (1, 6, 11, 18, 24, 27) and (1, 7, 11, 16, 24, 36), respectively. The two points marked as (1, 6, 9,12, 15, 18) and (1, 6 11, 18, 27, 31) represent the reported ground states by Chen^21^ and Garg^23^, respectively.

For C_55_B_5_, the ExCE energy versus the computational energy is shown in Fig. 6a. The putative ground state is (1, 7, 11, 24, 27). The five heteroatoms are located at the 5 apposite sites of 5 hexagon rings and make up of a pentagon which encloses a carbonic pentagon ring, with the similar pattern of the ground state of C_60-*n*_B_*n*_ for 2 ≤ *n* ≤ 4. The next preferred positions for boron atoms are (1, 7, 11, 32, 35), with a total energy of 0.32 eV higher. It was reported in ref.^23^ that the minimal energetic structure for C_55_B_5_ was (1, 7, 18, 51, 59). In contrast, this structure is found to be 253^rd^ in the stability ranking and higher in energy by 0.68 eV than our minimal energetic structure.

For C_54_B_6_, the putative lowest energy is from (1, 6, 11, 18, 24, 27) which is shown in Fig. 6b. In this isomer, 4 boron atoms are at the consecutive opposite sites and the other two are at the isolated opposite sites. The next most favorable configuration is (1, 7, 11, 16, 24, 36). Chen *et al*.^21^ reported that the global minimum structure was (1, 6, 9, 12, 15, 18), but from our result, this structure has a higher energy of 0.34 eV relative to our minimal energy and ranks 100^th^ in our ascending order list of the total energies. Garg *et al*.^23^ predicted the minimal energetic cage for C_54_B_6_ was (1, 6, 11, 18, 27, 31), now in our calculation, this structure rates the 340^st^ in the ranking of stability and is less stable by 0.52 eV with respect to our minimal energy structure.

## Summary

We have developed a nomenclature to enhance structural recognition and adopted an extended cluster expansion to describe the structural stabilities, which is good agreement with the results from the first-principles calculations. Unlike the conventional cluster expansion, the interaction parameters are derived from the enumeration of C_60-*n*_B_*n*_ (*n* = 1~4), where there are only 4 coefficients to be fitted for the composition consideration. Notably, we have found the stable isomers of C_57_B_3_, C_55_B_5_, and C_54_B_6_, which are energetically favored by at least 0.3 eV than the reported counterparts. With the symmetry operation matrices, the nomenclature can be applied for other binary/ternary systems, where the ground state structures are searched with the extended cluster expansion. Thus, our finding will be an effective complement to the first-principles calculations in materials science.

## Electronic supplementary material

Supplementary Information

Supplementary Dataset 1
