# Publisher Correction: An extended cluster expansion for ground states of heterofullerenes

**DOI:** 10.1038/s41598-019-51081-4

**Published:** 2019-10-09

**Authors:** Yun-Hua Cheng, Ji-Hai Liao, Yu-Jun Zhao, Xiao-Bao Yang

**Affiliations:** 1 Department of Physics, South China University of Technology, Guangzhou, 510640 People’s Republic of China; 2 Key Laboratory of Advanced Energy Storage Materials of Guangdong Province, South China University of Technology, Guangzhou, 510640 P. R. China

Correction to: *Scientific Reports* 10.1038/s41598-017-16469-0, published online 24 November 2017

In Figure 5a, bar 1 is missing results. The correct Figure 5 appears below as Figure 1.

**Figure 1:**
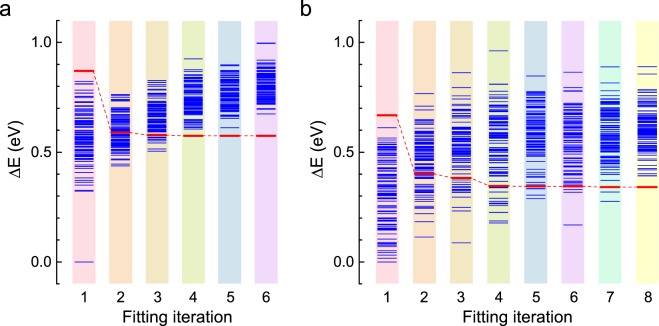
.

