# Supplementary material for: An extended cluster expansion for ground states of heterofullerenes
Source: Sci Rep. 2017 Nov 24;7:16211. doi: 10.1038/s41598-017-16469-0 (PMC5701149; doi:10.1038/s41598-017-16469-0)
Supplement: Supplementary file 1 — Supplementary Information [file 41598_2017_16469_MOESM1_ESM.docx]

Supplementary Information

**An extended cluster expansion for ground states of heterofullerenes**

Yun-Hua Cheng^1^, Ji-Hai Liao^1^, Yu-Jun Zhao^1, 2^, and Xiao-Bao Yang^1, 2*^

^1^Department of Physics, South China University of Technology, Guangzhou 510640,

People’s Republic of China

^2^Key Laboratory of Advanced Energy Storage Materials of Guangdong Province, South China University of Technology, Guangzhou 510640, P. R. China

1. **The C_60_ fullerene cage**

In our structural recognition method, we adopt the systematic numbering scheme recommended by IUPAC[^1^](#_ENREF_1). The coordinates and the sequence numbers (SNs) of each vertex of the relaxed C_60_ fullerene cage are listed in Table S1. The length unit of the coordinate is angstrom.

**Table S1 | Coordinates of the vertices of the C_60_ fullerene.**

| SN | x (Å) | y (Å) | z (Å) |
| --- | --- | --- | --- |
| 1 | 9.632 | 13.483 | 10.595 |
| 2 | 10.249 | 13.034 | 11.831 |
| 3 | 9.250 | 12.307 | 12.595 |
| 4 | 8.014 | 12.308 | 11.831 |
| 5 | 8.251 | 13.034 | 10.595 |
| 6 | 7.656 | 12.601 | 9.405 |
| 7 | 8.420 | 12.601 | 8.169 |
| 8 | 9.751 | 13.034 | 8.169 |
| 9 | 10.368 | 13.483 | 9.405 |
| 10 | 11.750 | 13.034 | 9.405 |
| 11 | 12.344 | 12.601 | 10.595 |
| 12 | 11.580 | 12.601 | 11.831 |
| 13 | 11.962 | 11.426 | 12.595 |
| 14 | 11.000 | 10.727 | 13.331 |
| 15 | 9.618 | 11.176 | 13.331 |
| 16 | 8.764 | 10.000 | 13.331 |
| 17 | 7.574 | 10.000 | 12.595 |
| 18 | 7.192 | 11.176 | 11.831 |
| 19 | 6.574 | 10.727 | 10.595 |
| 20 | 6.801 | 11.426 | 9.405 |
| 21 | 7.037 | 10.700 | 8.169 |
| 22 | 8.038 | 11.426 | 7.405 |
| 23 | 9.000 | 10.727 | 6.669 |
| 24 | 10.382 | 11.176 | 6.669 |
| 25 | 10.750 | 12.307 | 7.405 |
| 26 | 11.986 | 12.308 | 8.169 |
| 27 | 12.808 | 11.176 | 8.169 |
| 28 | 13.426 | 10.727 | 9.405 |
| 29 | 13.199 | 11.426 | 10.595 |
| 30 | 12.963 | 10.700 | 11.832 |
| 31 | 12.963 | 9.301 | 11.832 |
| 32 | 11.962 | 8.574 | 12.595 |
| 33 | 11.000 | 9.274 | 13.331 |
| 34 | 9.618 | 8.824 | 13.331 |
| 35 | 9.250 | 7.693 | 12.595 |
| 36 | 8.014 | 7.692 | 11.831 |
| 37 | 7.192 | 8.824 | 11.831 |
| 38 | 6.574 | 9.274 | 10.595 |
| 39 | 6.801 | 8.574 | 9.405 |
| 40 | 7.037 | 9.301 | 8.169 |
| 41 | 8.038 | 8.574 | 7.405 |
| 42 | 9.000 | 9.274 | 6.669 |
| 43 | 10.382 | 8.824 | 6.669 |
| 44 | 11.236 | 10.000 | 6.669 |
| 45 | 12.426 | 10.000 | 7.405 |
| 46 | 12.808 | 8.824 | 8.169 |
| 47 | 13.426 | 9.274 | 9.405 |
| 48 | 13.199 | 8.574 | 10.595 |
| 49 | 12.344 | 7.399 | 10.595 |
| 50 | 11.580 | 7.399 | 11.831 |
| 51 | 10.249 | 6.967 | 11.831 |
| 52 | 9.632 | 6.517 | 10.595 |
| 53 | 8.251 | 6.966 | 10.595 |
| 54 | 7.656 | 7.399 | 9.405 |
| 55 | 8.420 | 7.399 | 8.169 |
| 56 | 9.751 | 6.967 | 8.169 |
| 57 | 10.750 | 7.693 | 7.405 |
| 58 | 11.986 | 7.692 | 8.169 |
| 59 | 11.750 | 6.966 | 9.405 |
| 60 | 10.368 | 6.517 | 9.405 |

1. **Structure Recognition based on Numbering Matrix of C_60_**

C_60_ fullerene cage has 120 symmetry matrices (SMs). We summarized all the symmetry operations by a $60\times120$ numbering matrix (NM), as shown in the background of Fig. S1. (The full matrix is included in a Microsoft excel format file named numbering_matrix.xlsx, as one of the supplementary information files.) The NM is the base of our structure recognition method. In the NM, the *n*^th^ row lists the coincident atoms for the *n*^th^ atom under all the symmetry operations and the *n*^th^ column contains the corresponding coincident atoms for all the 60 atoms under the operation of the *n*^th^ SM. Taking the C_58_B_2_ isomers as an example, we adopt (1, 7) to identify the one with B atoms at para-positions of hexagon. According to the NM (the zoomed in 1^st^~15^th^ columns of the initial 15 rows high-lighted in Fig. S1a), the isomer with boron atoms at (1, 11) is same with the one of (1, 7) due to the symmetry operation. Similarly, the isomers with boron atoms at (2, 10) / (2, 14) / (3, 13) / (5, 8) / (6, 9) / (9, 12) / (12, 15) correspond to the same structure of (1, 7).


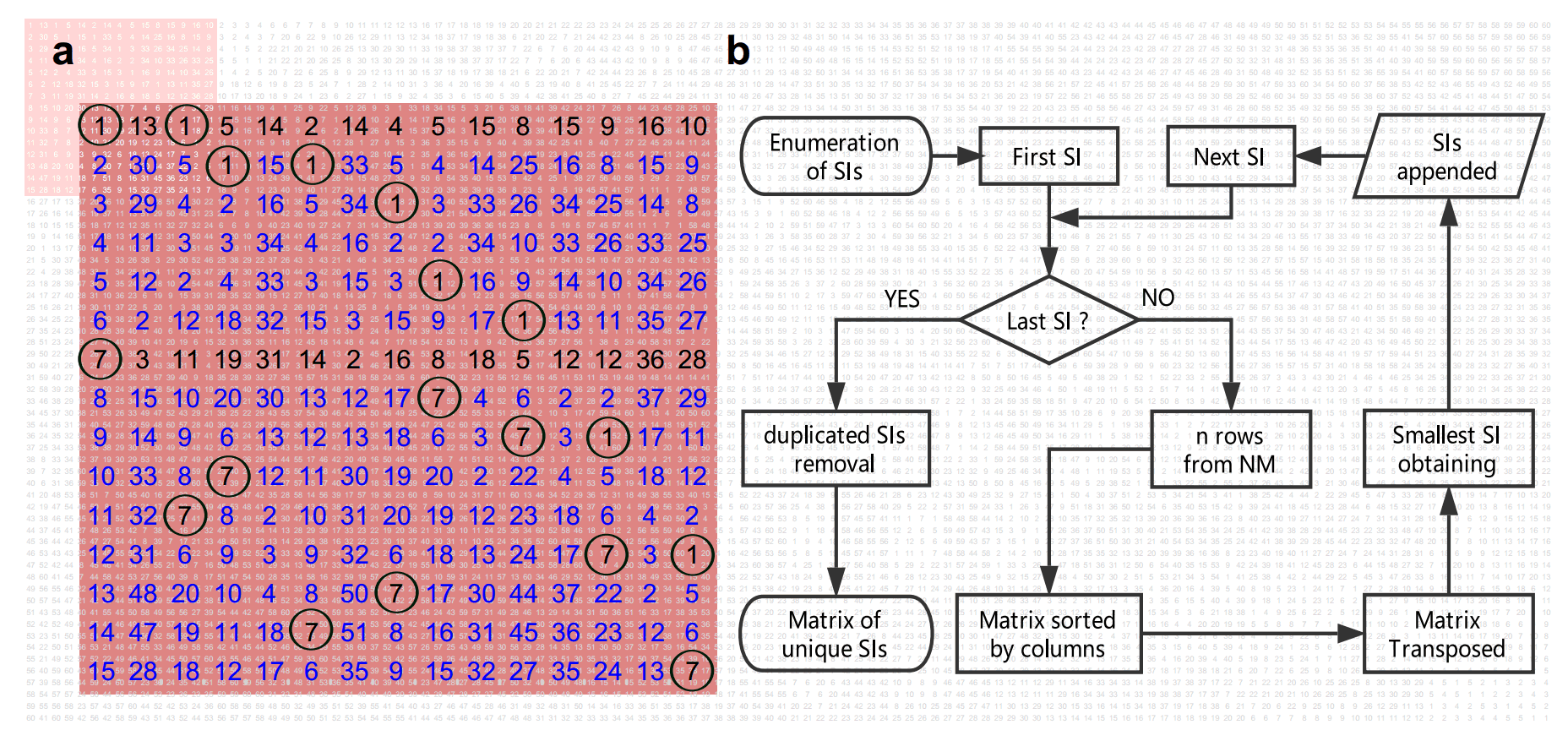


**Figure S1 | Diagram for the structure recognition scheme.** (**a**) Part of the numbering index matrix. The zoomed in part of the numbering matrix (NM) includes the 1^st^~15^th^ columns of the initial 15 rows from the NM. As an example shown in the zoomed part of the NM, the C_58_B_2_ isomers are equivalent if the sequence numbers of their boron substituted vertices are the numbers circled in any column, which are all denoted by (1, 7). All structural indexes (SIs) from any columns in the 1^st^ and 7^th^ rows (shown in black) are equivalent since they are all brought about by the symmetry operating on the isomer denoted by (1, 7). (**b**) Flowchart to obtain the structural indexes (SIs) of the inequivalent isomers of C_60-_*_n_*B*_n_* for a certain *n* value. Here in both (**a**) and (**b**), we make the NM as the background of the figure with a quite high resolution for readers to zoom into the graph to have a look. The data for NM are available in the Supplementary Dataset file.

The flow chart of our structure recognition method is shown in Fig. S1b. Any C_60-_*_n_*B*_n_* isomer is denoted by an index consisting of the ascending ordered SNs of the substituted vertices, *i.e.*${(\sigma}_{1},\sigma_{2},\ldots,\sigma_{n})$ which is called structural index (SI). Considering a certain structure denoted by SI, $n$ rows from NM make up a $n\times120$ matrix whose first column is just the transpose of the SI. We sort this matrix to make all the elements of any column in ascending order and then transpose it, which results in a new matrix called equivalent structure matrix (ESM). Each row of ESM is called as equivalent index (EI), implying that all structures denoted by these EIs are equivalent to that of the initial SI. We sort the rows of ESM in the ascending order based on the elements of each column from left to right, we then find the smallest EI and retain it as the ultimate SI for the structure. For C_60-_*_n_*B*_n_* with a certain *n* value, we can obtain the SIs for all the initial enumerated SIs, respectively After the duplicated removed, the remained SIs make up a matrix called as the inequivalent structure matrix (ISM). Each SI from the ISM denotes a unique isomer, which can serve as the identification (ID) of the corresponding C_60-_*_n_*B*_n_* isomer due to a consistent one-to-one match. The recursive algorithm can be used to gain ISM since the initial enumerated SIs of C_60-_*_n_*B*_n_* isomers can be derived from the ISM of C_60-_*_m_*B*_m_* where$m=n-1$.

From the structure recognition discussed above, we can determine whether two isomers are equivalent or not. For example, (14, 15, 16) and (14, 33, 34) correspond to the same structure, we prefer to choose their unique smallest EI, that is (1, 2, 3), to denote the structure.

1. **Enumerations of the isomers of C_60-_*_n_*B*_n_* heterofullerenes**

Using the structure recognition method, we enumerate the inequivalent isomers for C_60-_*_n_*B*_n_* heterofullerenes with variable boron concentration, as listed in Table S2 along with the corresponding combination number of$C_{60}^{n}$. Our results are in good agreement with the previous studies[^2^](#_ENREF_2)^,^ [^3^](#_ENREF_3)^,^ [^4^](#_ENREF_4). From the result, it can be inferred that the enumeration of isomers is about 1% of the corresponding combination number of$C_{60}^{n}$. On the other hand, those C_60-_*_n_*B*_n_* heterofullerenes for$n>4$ have so enormous isomers that it is impossible to conduct first principles calculations due to the expensive computation costs.

**Table S2 | Enumerations of the isomers of C_60-_*_n_*B*_n_* heterofullerenes.**

| Number of boron atoms | $C_{60}^{n}$ | Enumeration of isomers |
| --- | --- | --- |
| 1 | 60 | 1 |
| 2 | 1,770 | 23 |
| 3 | 34,220 | 303 |
| 4 | 487,635 | 4,190 |
| 5 | 5,461,512 | 45,718 |
| 6 | 50,063,860 | 418,470 |
| 7 | 386,206,920 | 3,220,218 |
| 8 | 2,558,620,845 | 21,330,558 |
| 9 | 14,783,142,660 | 123,204,921 |
| 10 | 75,394,027,566 | 628,330,629 |

1. **The detailed data from the fitting for C_55_B_5_ and C_54_B_6_**

Follow those steps of the flow chart of the extend cluster expansion (ExCE) method, we have made the prediction for the energies of the selected isomers of C_55_B_5_ and C_54_B_6_ heterofullerenes. The data of the fitting steps which are 6 and 8 for C_55_B_5_ and C_54_B_6_, respectively, are listed in Table S3 and Table S4 for C_55_B_5_ and C_54_B_6_, respectively. The columns, from left to right, are the sequence number of the fitting step, coefficients and the corresponding cross-validation (CV) scores, the number of isomers and the number of the new added structures whose energies are among the lowest 100 structures for the fitting steps, respectively.

**Table S3 | Detailed data of the fitting for C_55_B_5_.**

| Fitting step | $c_{1}$ | $c_{2}$ | $c_{3}$ | $c_{4}$ | CV (eV) | Number of isomers | New added |
| --- | --- | --- | --- | --- | --- | --- | --- |
| 1 | 0.956 | 0.570 | 0.353 | -0.041 | 0.051 | 100 | 100 |
| 2 | 0.956 | 0.556 | 0.383 | 0.017 | 0.054 | 200 | 44 |
| 3 | 0.955 | 0.543 | 0.395 | 0.063 | 0.058 | 300 | 9 |
| 4 | 0.958 | 0.571 | 0.416 | 0.094 | 0.061 | 400 | 1 |
| 5 | 0.959 | 0.574 | 0.422 | 0.112 | 0.060 | 500 | 0 |
| 6 | 0.960 | 0.585 | 0.429 | 0.132 | 0.060 | 600 | 0 |

**Table S4 | Detailed data of the fitting for C_54_B_6_.**

| Fitting step | $c_{1}$ | $c_{2}$ | $c_{3}$ | $c_{4}$ | CV (eV) | Number of isomers | New added |
| --- | --- | --- | --- | --- | --- | --- | --- |
| 1 | 0.976 | 0.693 | 0.532 | 0.003 | 0.131 | 100 | 100 |
| 2 | 0.975 | 0.680 | 0.520 | 0.027 | 0.126 | 200 | 33 |
| 3 | 0.972 | 0.660 | 0.488 | 0.037 | 0.127 | 300 | 16 |
| 4 | 0.970 | 0.658 | 0.447 | 0.011 | 0.132 | 400 | 13 |
| 5 | 0.971 | 0.672 | 0.448 | 0.017 | 0.129 | 500 | 4 |
| 6 | 0.966 | 0.620 | 0.412 | 0.033 | 0.129 | 600 | 3 |
| 7 | 0.963 | 0.592 | 0.399 | 0.029 | 0.128 | 700 | 2 |
| 8 | 0.963 | 0.587 | 0.399 | 0.031 | 0.124 | 800 | 0 |

1. **Lists of the isomers of C_60-_*_n_*B*_n_* heterofullerenes**

The structure indexes (SIs) of the 23 isomers of C_58_B_2_, and the 80 lowest energetic isomers of C_60-_*_n_*B*_n_* for $3\leq n\leq6$, are listed in Table S5, each column of which is in the ascending order of energy. The numbers in first column from the left represent the energetic ranks of the corresponding isomers.

**Table S5 | A List of the structural indexes of the isomers of C_60-_*_n_*B*_n_* heterofullerene.**

| Rank | C_58_B_2_ | C_57_B_3_ | C_56_B_4_ | C_55_B_5_ | C_54_B_6_ |
| --- | --- | --- | --- | --- | --- |
| 1  2  3  4  5  6  7  8  9  10  11  12  13  14  15  16  17  18  19  20  21  22  23  24  25  26  27  28  29  30  31  32  33  34  35  36  37  38  39  40  41  42  43  44  45  46  47  48  49  50  51  52  53  54  55  56  57  58  59  60  61  62  63  64  65  66  67  68  69  70  71  72  73  74  75  76  77  78  79  80 | 1,7  1,23  1,41  1,50  1,52  1,60  1,3  1,16  1,33  1,56  1,49  1,13  1,32  1,57  1,9  1,35  1,31  1,6  1,24  1,15  1,14  1,34  1,2 | 1,7,11  1,7,28  1,6,11  1,7,49  1,7,32  1,7,46  1,7,35  1,7,51  1,7,48  1,7,36  1,3,7  1,7,34  1,7,14  1,7,33  1,7,30  1,6,18  1,7,31  1,7,16  1,7,37  1,7,17  1,3,11  1,7,29  1,7,15  1,7,27  1,7,18  1,7,50  1,7,13  1,7,26  1,23,28  1,23,36  1,3,23  1,6,16  1,3,28  1,6,28  1,23,50  1,23,32  1,3,47  1,3,41  1,23,31  1,16,54  1,41,48  1,23,35  1,16,28  1,14,47  1,14,23  1,32,41  1,23,34  1,16,23  1,32,39  1,6,35  1,3,43  1,6,52  1,23,33  1,3,55  1,24,50  1,6,48  1,14,41  1,6,50  1,23,37  1,16,56  1,16,41  1,3,45  1,33,41  1,6,23  1,14,58  1,14,56  1,15,28  1,15,23  1,31,38  1,6,46  1,31,42  1,3,29  1,6,59  1,14,54  1,31,54  1,13,23  1,3,39  1,31,41  1,3,56  1,14,28 | 1,7,11,24  1,7,32,35  1,6,11,18  1,7,49,52  1,7,16,36  1,7,34,37  1,7,28,31  1,7,48,58  1,7,14,31  1,7,17,35  1,7,33,51  1,3,11,13  1,3,7,17  1,7,13,32  1,7,15,18  1,3,7,13  1,6,11,16  1,7,28,33  1,7,34,50  1,7,11,44  1,7,11,56  1,7,11,43  1,6,11,35  1,7,11,34  1,7,11,49  1,6,18,28  1,7,46,51  1,7,30,35  1,7,14,47  1,3,7,36  1,7,14,58  1,3,7,47  1,7,34,59  1,7,29,59  1,7,11,51  1,7,11,57  1,7,16,56  1,7,32,37  1,7,11,35  1,3,43,55  1,3,47,59  1,3,29,48  1,3,7,31  1,6,18,48  1,6,18,23  1,3,23,40  1,6,11,44  1,6,18,50  1,6,11,50  1,7,32,58  1,3,7,43  1,7,34,46  1,7,11,15  1,3,7,12  1,7,28,51  1,7,28,43  1,6,11,59  1,7,11,42  1,7,28,35  1,3,11,36  1,7,11,50  1,7,28,53  1,7,11,52  1,7,29,36  1,7,32,53  1,7,11,32  1,7,30,58  1,7,16,54  1,6,18,41  1,7,16,52  1,3,42,56  1,3,7,48  1,7,14,56  1,7,11,31  1,7,15,55  1,3,11,31  1,7,17,52  1,7,15,46  1,6,11,27  1,6,11,51 | 1,7,11,24,27  1,7,11,32,35  1,7,11,51,59  1,7,11,49,52  1,6,11,18,27  1,7,11,43,55  1,7,11,43,46  1,7,11,16,36  1,7,11,24,36  1,7,11,24,49  1,7,11,44,58  1,7,11,34,37  1,6,11,24,27  1,7,11,24,35  1,7,11,24,51  1,7,11,24,32  1,7,11,33,51  1,3,7,24,27  1,7,11,42,56  1,7,11,41,57  1,6,11,15,18  1,7,11,24,33  1,3,7,13,24  1,3,7,47,59  1,6,11,16,36  1,3,7,53,56  1,7,14,47,59  1,3,7,17,24  1,3,7,46,49  1,7,11,24,31  1,7,11,16,24  1,7,11,24,48  1,6,11,42,56  1,6,11,53,56  1,6,12,15,18  1,6,11,51,59  1,7,11,24,37  1,6,11,32,35  1,7,16,28,36  1,6,11,43,55  1,7,11,16,27  1,7,30,53,56  1,3,11,24,27  1,6,11,18,56  1,3,7,13,32  1,3,7,36,39  1,7,16,47,59  1,6,11,18,57  1,3,7,49,52  1,6,11,18,44  1,6,11,18,43  1,7,17,46,49  1,6,11,18,59  1,7,28,34,37  1,6,11,17,35  1,6,11,34,50  1,6,11,52,55  1,3,11,36,39  1,6,11,18,28  1,6,11,36,39  1,7,28,33,51  1,7,11,14,31  1,6,11,18,42  1,3,7,43,46  1,7,14,43,46  1,7,16,36,46  1,7,28,53,56  1,7,16,36,43  1,7,29,53,56  1,7,14,46,49  1,3,7,25,45  1,7,16,36,45  1,3,7,23,40  1,6,11,18,35  1,7,28,37,54  1,7,17,47,59  1,7,16,36,47  1,6,11,16,18  1,3,11,42,56  1,6,11,18,52 | 1,6,11,18,24,27  1,7,11,16,24,36  1,6,11,18,42,56  1,7,11,24,49,52  1,7,11,24,33,51  1,7,16,36,43,46  1,6,11,18,53,56  1,7,11,24,32,35  1,6,11,18,43,55  1,6,11,18,28,31  1,7,11,24,27,35  1,7,11,16,27,36  1,7,11,49,52,55  1,6,11,18,52,55  1,7,11,16,24,27  1,3,7,17,24,27  1,6,11,18,45,57  1,7,16,30,36,47  1,7,16,36,46,49  1,7,15,18,47,59  1,3,7,13,32,35  1,3,6,11,13,18  1,6,11,16,18,36  1,6,11,18,51,59  1,6,11,16,24,27  1,6,11,18,32,35  1,6,11,16,36,39  1,3,7,11,13,24  1,7,11,24,48,58  1,3,7,17,46,49  1,3,7,13,53,56  1,7,28,31,37,54  1,6,11,18,36,39  1,7,14,31,53,56  1,7,11,14,24,31  1,6,11,18,47,59  1,6,11,18,41,57  1,7,11,14,24,27  1,3,7,13,24,27  1,3,7,13,49,52  1,3,7,13,23,40  1,7,14,31,43,55  1,6,11,16,43,55  1,7,16,36,48,58  1,3,7,13,16,36  1,7,11,33,51,59  1,7,16,36,45,57  1,3,7,10,17,28  1,3,11,13,42,56  1,3,7,11,17,27  1,3,7,11,17,24  1,6,11,16,53,56  1,7,14,31,36,39  1,3,7,17,44,58  1,7,11,14,27,31  1,7,14,31,38,53  1,7,14,31,37,54  1,3,7,13,28,31  1,3,7,46,49,52  1,3,7,13,51,59  1,3,11,13,23,40  1,6,11,18,23,40  1,3,7,24,27,47  1,7,11,32,35,44  1,7,15,18,53,56  1,3,7,11,24,27  1,3,7,13,25,45  1,7,13,32,43,55  1,3,7,17,43,46  1,6,11,18,48,58  1,3,7,17,23,40  1,3,7,17,47,59  1,7,16,28,31,36  1,7,11,16,36,39  1,7,11,24,46,49  1,7,14,31,45,57  1,3,7,47,49,52  1,3,11,13,21,42  1,7,17,35,48,58  1,6,11,16,42,56 |

1. **Details for the first-principles calculations**

The first-principles calculations in this paper are performed using the Perdew-Burke-Ernzerhof electron exchange-correlation functional within generalized gradient approximation (PBE-GGA)[^5^](#_ENREF_5). The projector augmented wave (PAW)[^6^](#_ENREF_6)^,^ [^7^](#_ENREF_7) pseudopotentials implemented were adopted in the Vienna Ab-initio Simulation Package (VASP)[^8^](#_ENREF_8)^,^ [^9^](#_ENREF_9)^,^ [^10^](#_ENREF_10). Structures were optimized using conjugate gradient algorithm and the residual forces were less than 0.02 eV/Å. A simple cubic shell of 20 Å was adopted to avoid any significant spurious interactions with periodically repeated images and the plane wave cutoff energy was set to be 520 eV. As a test, we found that the optimized boron doped C_60_ cages still appeared to be particularly stable just as the corresponding pure carbon fullerenes, which is in agreement with the experimental results[^11^](#_ENREF_11). The lengths of single and double bonds of C_60_ fullerene were found to be 1.45 Å and 1.39 Å, respectively. All these calculations were in agreement with the experimental values[^12^](#_ENREF_12) and the pervious theoretical calculations[^13^](#_ENREF_13)^,^ [^14^](#_ENREF_14).

**References**

1. Cozzi F, Powell WH, Thilgen C. Numbering of fullerenes - (IUPAC Recommendations 2005). *Pure and Applied Chemistry* **77**, 843-923 (2005).

2. Shinsaku F. Soccerane Derivatives of Given Symmetries. *Bulletin of the Chemical Society of Japan* **64**, 3215-3223 (1991).

3. Balasubramanian K. Enumeration of chiral and positional isomers of substituted fullerene cages (C_20_-C_70_). *The Journal of Physical Chemistry* **97**, 6990-6998 (1993).

4. Babic D, Doslic T, Klein DJ, Misra A. Kekulenoid addition patterns for fullerenes and some lower homologs. *Bulletin of the Chemical Society of Japan* **77**, 2003-2010 (2004).

5. Perdew JP, Burke K, Ernzerhof M. Generalized Gradient Approximation Made Simple. *Physical review letters* **77**, 3865-3868 (1996).

6. Blöchl PE. Projector augmented-wave method. *Physical Review B* **50**, 17953-17979 (1994).

7. Kresse G, Joubert D. From ultrasoft pseudopotentials to the projector augmented-wave method. *Physical Review B* **59**, 1758-1775 (1999).

8. Kresse G, Hafner J. Ab initio molecular dynamics for liquid metals. *Physical Review B* **47**, 558-561 (1993).

9. Kresse G, Furthmüller J. Efficient iterative schemes for ab initio total-energy calculations using a plane-wave basis set. *Physical Review B* **54**, 11169-11186 (1996).

10. Kresse G, Furthmüller J. Efficiency of ab-initio total energy calculations for metals and semiconductors using a plane-wave basis set. *Computational Materials Science* **6**, 15-50 (1996).

11. Guo T, Jin C, Smalley RE. Doping bucky: formation and properties of boron-doped buckminsterfullerene. *The Journal of Physical Chemistry* **95**, 4948-4950 (1991).

12. Hedberg K*, et al.* Bond lengths in free molecules of buckminsterfullerene, C_60_, from gas-phase electron diffraction. *Science (New York, NY)* **254**, 410-412 (1991).

13. Zhang QM, Yi J-Y, Bernholc J. Structure and dynamics of solid C_60_. *Physical review letters* **66**, 2633-2636 (1991).

14. Garg I, Sharma H, Dharamvir K, Jindal VK. Substitutional Patterns in Boron Doped Heterofullerenes C_60-n_B_n_ (n = 1-12). *Journal of Computational and Theoretical Nanoscience* **8**, 642-655 (2011).
